# Clinical Effectiveness of Continuous Pressure Monitoring Systems for Pressure Ulcer Prevention: A Scoping Review

**DOI:** 10.1111/iwj.71010

**Published:** 2026-08-11

**Authors:** Rashedul Hoque, Nicci Aylward‐Wotton, Sara Noman, Silvia Caggiari, Joanne Lloyd, Reg Taylor, Caroline Searight, Euan Sadler, Bridie Kent, Rebecca Hardwick, Davide Filingeri, Liudi Jiang, Stephen Duckworth, Peter R. Worsley

**Affiliations:** ^1^ School of Health Sciences, University of Southampton Southampton UK; ^2^ Cornwall Partnership NHS Foundation Trust Bodmin UK; ^3^ Faculty of Engineering and Physical Sciences, University of Southampton Southampton UK; ^4^ Transforming Community Health Patient and Public Involvement and Engagement (PPIE) Group Southampton UK; ^5^ The University of Plymouth Centre for Innovations in Health and Social Care, University of Plymouth Plymouth UK

**Keywords:** clinical evaluation, continuous pressure monitoring, mixed methods synthesis, pressure ulcers

## Abstract

There are many circumstances where individuals with limited mobility are exposed to prolonged postures increasing the risk of pressure ulcers. Technologies have been developed to monitor posture, mobility and pressure exposure; however, their effectiveness in different clinical settings is unknown. The aim of this scoping review was to assess clinical studies using continuous pressure monitoring for the prevention and/or treatment of pressure ulcers. A scoping review of the literature was conducted using the PRISMA‐ScR framework. Clinical‐based studies were included which used continuous pressure monitoring for assessment and treatment over a minimum of a 2‐h period. The outcomes included quantitative measures such as pressure distribution and mobility data, qualitative insights relating to patient comfort and acceptability, and the perceptions of healthcare staff regarding usability and clinical integration of the technology. Twenty‐four studies were identified and included in the scoping review, conducted across eight countries spanning three continents. Most studies were undertaken in a hospital setting (67%, *n* = 16). Following review of the included papers, five core themes were identified: clinical outcomes, pressure metrics, posture and mobility, nurse feedback and patient experience. There was high heterogeneity in study design, outcome measures and different risk of bias limited the scope to synthesise the outcomes. Collectively, the studies indicate that CPM can enhance awareness of interface pressures, support clinical decision‐making and inform repositioning strategies for individuals at risk of tissue damage. Reported benefits include greater staff confidence and patient engagement. However, consistent reductions in ulcer incidence have yet to be demonstrated, with larger trials with standardised outcomes required.

## Highlights

- Continuous pressure monitoring has been used in different care settings to assess pressure ulcer risk and mobility status.
- There is no consensus on common outcome measures associated with CPM technologies limiting comparison.
- Quantitative data reveals a limited effect of CPM on improvement in pressure parameters and incidence of pressure ulcers.
- Qualitative data demonstrated a positive impact on patients and healthcare workers acting as a biofeedback tool.
- There is a need for more standardised assessment and reporting of CPM in care settings, including human factors associated with implementation.

## 1 Introduction

Technologies that can both monitor and promote active mobility, assisting those who are frail and identify detrimental health signs are critical to our future digitally enabled care provision [1]. Individuals residing in home and social care settings often have limited mobility due to aging, and comorbidities. One of the health impacts from impaired mobility are pressure ulcers (PUs). These are defined as localised injury to the skin and/or underlying tissue usually over a bony prominence, as a result of prolonged pressure or pressure in combination with shear [2]. PUs represent a major burden to populations worldwide and despite increased awareness, they contribute to a chronic wound care cost of £8 billion p.a. in the UK [3], and of $22 billion p.a. in the USA [4].

Prevention strategies currently focus on pressure relief via periodic repositioning and pressure redistribution. This is typically achieved through support surface selection and self‐evoked movements, or where mobility is limited, by clinicians and carers who manually adjust the individual's posture. However, this can be time consuming and labour intensive [5]. Although national guidelines provide evidence‐based recommendations on PU prevention, assessment, repositioning frequency and support surface use, clinical implementation remains variable [6]; practice still relies on professional judgement and locally preferred risk tools rather than a single mandated standard. Accordingly, in many community settings where resources are limited, the recommended frequency and magnitude of movements are not followed [7], or delivered effectively [8]. To address this urgent gap, safe and appropriate technologies are required to support self‐management and timely interventions. This aligns to new policy from health and social care providers around the world, where technology‐enabled personalised care is seen as a critical part of future innovation to meet the demands of an aging population [9, 10, 11].

Guidelines on PU prevention and treatment suggest a 2‐step approach whereby patients who are admitted to a care service (hospital or care home) are screened, then a formal risk assessment is undertaken [12]. In current practice this typically involves observations from healthcare professionals and risk assessment tools, which are often not supported by technology. As care provision transitions to technology‐enabled assessment and monitoring, there is a role for innovations to support care decisions, including skin assessment and the evaluation of posture, mobility and pressure risk [13]. However, with the emergence of these technologies there needs to be clear evidence on their effectiveness, enablement of person centre care and role in supporting healthcare workers in their routine practice.

An example of existing technology to aid clinical decision‐making is pressure‐sensing arrays, for example, seat or bed maps, which are used to assess individuals in sitting or lying postures [14]. These commercial systems provide visual feedback of pressure ‘hot spots’ to optimise individual repositioning and prescribe pressure‐relieving cushions and mattresses [15]. A meta‐analysis showed that the risk of developing new PUs with the use of monitoring devices may be 88% lower than that without the use of monitoring devices [13]. Multiple sensors are available that can detect body positions; however, with varying accuracy. Mostly inertial sensors have been clinically investigated demonstrating that a sensor equipped with a body‐turn warning system can reduce PU occurrence and increase turn compliance [16]. However, these body‐worn inertial sensors lack the critical information regarding the interaction between the person and the support surface, which is fundamental to the mechanisms which cause skin and underlying tissue damage [2].

In recent years, technology has been modified to enable continuous pressure monitoring (> 24 h), where studies have used this technology in hospital [14] and community settings [17]. Specific features within the pressure data have been shown to be both sensitive and specific to postural movements and Machine Learning (ML) has been employed to efficiently analyse movement patterns [18]. This was successfully translated to assess vulnerable patients during prolonged periods of lying and sitting [19, 20]. The clinical translation of these technologies has been previously limited to date due to the complexity and cost of the equipment [21], with new technologies being established to address these barriers to implementation. Continuous pressure mapping systems have been modified recently to manage the complexity and volume of data, which inevitably includes redundancy [22]. Current CPM systems vary in the type, resolution and performance of sensor technologies [16]. Critically, there is little consensus regarding the clinical translation of CPM and what the strengths of evidence there are for its effectiveness in influencing care decisions and how CPM is effective in preventing PUs.

This scoping review aims to synthesise evidence from literature where CPM has been used to assess and treat patients at risk or who have PUs in community and hospital settings. The objectives are to define how CPM systems are currently implemented and what outcomes have been reported for the prevention and treatment of PUs. Critically the scoping review aims to answer the following question:

How is continuous pressure monitoring being used to support care delivery for the prevention and treatment of PUs in care settings?

## 2 Methods

This study followed a scoping review approach to provide a structured synthesis of published evidence in this field. The purpose of this review was to evaluate the clinical applications of CPM systems in the prevention and management of PUs among adult patients.

### 2.1 Eligibility Criteria

Eligible studies were original research, written in English, that investigated CPM in a clinical setting. To be considered as CPM, the monitoring period had to extend for at least 2 h; shorter, transient pressure mapping or static support surface evaluations were excluded. At least one patient had to be included, and conference abstracts, editorials, opinion papers and protocols were not eligible.

### 2.2 Information Sources

To identify potentially relevant documents, the following bibliographic databases were searched from July 2025 to August 2025 across the following databases: Cochrane Central Register of Controlled Trials (CENTRAL), Ovid MEDLINE, Ovid MEDLINE (In‐Process &and Other Nonindexed Citations), Ovid EMBASE, EBSCO CINAHL Plus and PubMed. The search strategies were drafted by an experienced librarian and further refined through team discussion. The final search strategy for MEDLINE can be found below. The final search results were exported into EndNote, and duplicates were removed.

#### 2.2.1 Search Strategy

The search combined synonyms for PUs and related monitoring technologies. Key terms included:

- PU terminology (#1: ‘pressure ulcer*’ OR ‘pressure damage*’ OR ‘pressure injur*’ OR ‘pressure sore*’ OR ‘decubitus ulcer*’ OR ‘bed sore*’ OR ‘decubitus’)
- Monitoring and sensing terminology (#2–#6: ‘pressure mapping’, ‘continuous pressure monitor*’, ‘interface pressure’, ‘biofeedback system*’, ‘machine learning’, ‘mobility monitor*’, etc.)
- The final strategy combined #1 AND (#2 OR #3 OR #4 OR #5 OR #6).

### 2.3 Selection of Sources of Evidence

The outcome of interest was broad, encompassing the role of CPM in supporting clinical practice for PU prevention. This included quantitative measures such as pressure distribution and mobility data, qualitative insights relating to patient comfort and acceptability, and the perceptions of healthcare staff regarding usability and clinical integration of the technology. The PRISMA extension for scoping reviews (PRISMA‐ScR) was used to inform the design [23]. Titles and abstracts were manually screened against the eligibility criteria. Full texts of potentially relevant studies were obtained and assessed independently by three reviewers (RH, NAW, SN). We resolved disagreements on study selection and data extraction by consensus and discussion with other reviewers if needed.

### 2.4 Data Items

Data were extracted into a literature matrix capturing study authors, year of publication, study design, setting, population, CPM system used and key outcomes. We abstracted data on article characteristics (e.g., country of origin, funder), engagement characteristics and contextual factors (e.g., type of CPM user, type of CPM engagement activity, frequency and intensity of CPM engagement), barriers and facilitators to CPM implementation and results of any formal assessment, for example, prevalence or incidence of PUs.

### 2.5 Critical Appraisal

A broad inclusion strategy was adopted to encompass the full range of clinical evidence available, with additional notes recorded on the relevance and clinical applicability of findings. Due to the scoping nature of the literature review, no formal risk of bias assessment was conducted.

### 2.6 Synthesis of Results

We grouped the studies by the types of CPM evaluations, and summarised the type of settings, populations and study designs for each study, along with the measures used and broad findings. A content analysis approach was then undertaken, whereby reported outcomes were identified and studies were grouped into emergent thematic categories, with this interpretive process undertaken by multiple reviewers to enhance reliability. This interpretive review process enabled comparison across diverse studies and informed the thematic analysis of emerging concepts, methodological patterns and lessons relevant to clinical translation of CPM technologies.

## 3 Results

### 3.1 Selection of Sources of Evidence

The database search yielded 3914 records, with an additional six identified through trial registries. From the six records retrieved through clinical trial registries, five used pressure monitoring systems but were all excluded for not meeting criteria: four had no posted results or involved healthy participants or monitoring under 2 h [24, 25, 26, 27] and one completed trial [28] included only brief mapping in healthy volunteers. After removal of duplicates (*n* = 1957), 1963 unique records were screened by title and abstract. Of these, 1840 were excluded as irrelevant, leaving 123 full texts for retrieval. Seven could not be accessed in full, resulting in 116 articles assessed for eligibility.

**FIGURE 1:**
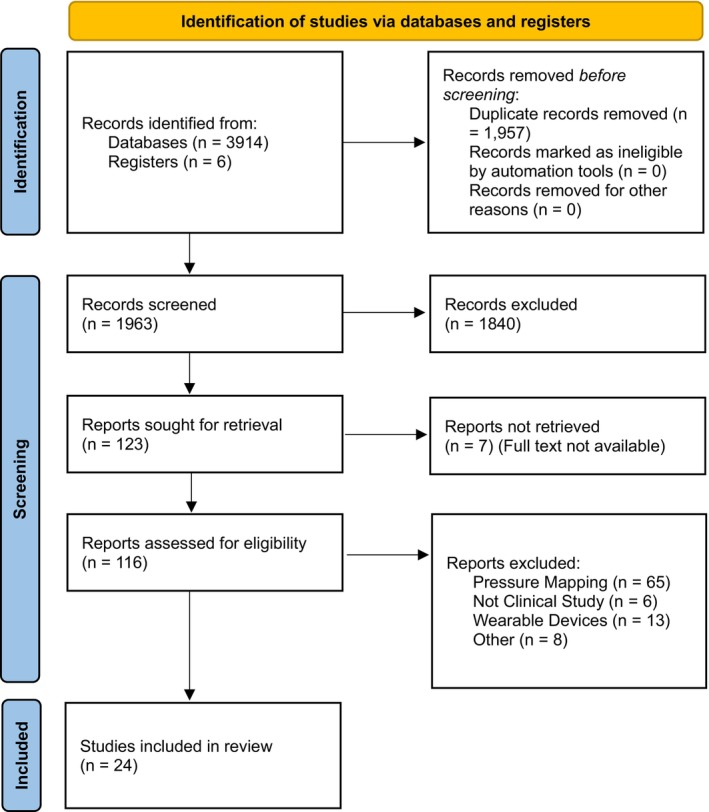
PRISMA flow diagram for study selection.

From these, 92 articles were excluded: 65 described transient pressure mapping of less than 2 h, 13 focused on wearable devices, six were not clinical studies and eight classified as ‘other’, consisting of reviews, meta‐analyses or background reports. A total of 24 studies met the inclusion criteria and were included in this review. The PRISMA flow diagram is shown in Figure 1, providing a summary of the study selection process. The characteristics of all 24 included studies are presented in Table 1.

**TABLE 1:** Characteristics of included studies.

First author	Year	Country	Study design	Setting	Population group
C. Ho [29]	2023	United States	Randomised controlled trial	Hospital	86 patients with mobility impairment
D. F. Shih [30]	2020	Taiwan	Experimental clinical study with engineering validation	Hospital	47 cardiac surgery patients
E. B. M. Sving [31]	2023	United States	Quasi‐experimental, nonrandomised intervention trial	Hospital	116 Adults undergoing general surgery
H. M. Gammon [32]	2016	Germany	Prospective case series	Clinic/rehabilitation unit	101 inpatients
K. Sakai [33]	2009	Japan	Observational study	Hospital	30 ICU participants
K. Tatsuta [34]	2024	Japan	Prospective cohort study	Hospital	37 patients undergoing laparoscopic colorectal surgery
K. Woo [35]	2022	United States	Prospective descriptive feasibility study	Hospital	104 patients at risk of pressure injury
L. Gunningberg [36]	2018	Sweden	Descriptive qualitative study	Hospital	50 healthcare professionals
L. Gunningberg [37]	2017	Sweden	Randomised controlled trial	Hospital	190 patients
L. Hultin [38]	2019	United States	Descriptive study	Hospital	31 orthopaedic patients
L. Hultin [39]	2017	United States	Descriptive study	Hospital	40 assistant nurses, 12 elderly residents
M. Hubli [40]	2021	United Kingdom	Prospective cross‐sectional prepost pilot study	Clinic/rehabilitation unit	Nine participants with SCI
M. J. Grap [41]	2017	United states	Descriptive, longitudinal study	Hospital	132 general inpatients
N. Aylward‐Wotton [17]	2025	England	Mixed methods case study design	Community	44 community patients
O. Chenu [42]	2013	France	Preliminary longitudinal case study	Community	One male paraplegic volunteer
R. Behrendt [43]	2014	United States	Prospective controlled study	Hospital	422 inpatients
R. G. Scott [44]	2014	United States	Interventional clinical study	Hospital	10 patients in long term care facility
S. Al‐Majid [45]	2022	United States	Descriptive, repeated‐measures study	Community	150 adult surgical patients (129 included in final analysis)
S. Caggiari [46]	2024	England	Secondary analysis; observational study	Community	22 community residents with existing pressure ulcers
S. Caggiari [19]	2021	United Kingdom	Observational feasibility and algorithm validation	Clinic/rehabilitation unit	Two individuals with SCI
S. Fryer [47]	2023	Netherlands	Pilot observational clinical study	Clinic/rehabilitation unit	20 inpatients with SCI
S. M. Motamedi [48]	2012	Canada	Pilot study; prospective interventional	Hospital	12 inpatients
T. L. Vos‐Draper [49]	2023	Canada	Longitudinal, repeated‐measures pilot intervention study	Clinic/rehabilitation unit	23 adult wheelchair users with complete SCI
W. Ocampo [50]	2022	United States	Secondary analysis of randomised controlled trial data	Hospital	525 patients and 125 healthcare professionals

### 3.2 Characteristics of Sources of Evidence

The studies included in the review are described in Table 1, together with a description of study design, location and population group. The 24 included studies were conducted across eight countries spanning three continents (North America, Europe and Asia). The largest proportion of studies (25%, *n* = 6) originated from the United States [32, 41, 43, 44, 45, 49]; followed by Sweden (~21%, *n* = 5) [31, 36, 37, 38, 39]; Canada [29, 35, 48, 50] and the United Kingdom (~17%, *n* = 4 each) [17, 19, 46, 47]; Japan (~8%, *n* = 2) [33, 34] and single studies in France [42], Switzerland [40] and Taiwan [30].

Most studies were undertaken in a hospital setting (67%, *n* = 16) [29, 30, 31, 33, 34, 35, 36, 37, 38, 39, 41, 43, 44, 45, 48, 50], including acute medical, surgical and intensive‐care settings. A smaller number were undertaken in clinic or rehabilitation units (21%, *n* = 5) [19, 32, 40, 47, 49]. The remaining studies were conducted in a community environment (13%, *n* = 3), such as long‐term residential care or private homes [17, 42, 46].

Table 2 provides a summary of the results of individual sources of evidence and the themes in which the studies were included in the evidence synthesis.

**TABLE 2:** Summary of the study design, CPM systems and core outcomes.

Authors	Study design/method	CPM system used	Duration of pressure sensing	Themes	Bed/seating	Outcome summary
Al‐Majid S and Vuncanon B and Kiyohara M and Rakovski C	Observational repeated‐measures design	XSENSOR X3 Pro 8	Unknown	Biofeedback, clinical/patient outcomes	Operating table	The percentage of time during which the interface pressure exceeded 32 mmHg at the scapulae, interscapular area and sacral area was 70%, 70% and 90%, respectively. Body mass index, length of surgery and intraoperative position were major predictors of increased pressure.
Aylward‐Wotton N, Worsley P, Kent B	Mixed methods case study design	ForeSitePT, Xsensor	24–72 h	Biofeedback, clinical/patient outcomes	Bed	Both nurses and carers valued the data generated by the technology. This resulted in an increase in patient‐centred care, an increase in patient empowerment, the ability to influence patient decision making and equipment choices. However, using CPM in a real‐world community setting stretched the technology beyond its original design, which was intended for hospital use.
Caggiari S and Aylward‐Wotton N and Kent B and Worsley PR	Secondary analysis of data from a quality improvement project (PROMISE), observational study	ForeSite PT pressure mapping system (XSENSOR Technology Corp)	5 h—5 days (median 21.3 h)	Biofeedback	Bed	High intersubject variability in posture, mobility and pressure exposure. 17/22 spent > 60% time (> 2 h continuously) in static postures. Frequent prolonged exposure to harmful peak pressure gradients (> 60 mmHg/cm). Mobility (frequency of posture changes) generally low, associated with prolonged static postures.
Caggiari, S and Worsley, P R and Fryer, S L and Mace, J and Bader, D L	Observational feasibility study and algorithm validation	ForeSite PT pressure mapping system (XSENSOR Technology Corp)	Testing phase duration not explicitly stated; long‐term trends assessed; real‐time capability demonstrated	Biofeedback, clinical/patient outcomes	Bed and Wheelchair	Demonstrated feasibility of combining pressure mapping and CNN to detect and classify posture in vulnerable SCI patients, supporting personalised care and clinical decision‐making.
Chenu, Olivier and Vuillerme, Nicolas and Bucki, Marek and Diot, Bruno and Cannard, Francis and Payan, Yohan	Preliminary longitudinal case study	TexiCare: 32Ã—32 textile pressure sensor matrix (1024 sensors)	6 months of continuous home monitoring	Biofeedback, clinical/patient outcomes	Wheelchair	CPM revealed infrequent and ineffective relief manoeuvres, asymmetrical posture while watching TV and high ischial pressures at day's end. Enabled posture adjustments and improved cushion use strategy.
Fryer, Sarah and Caggiari, Silvia and Major, Denise and Bader, Dan L and Worsley, Peter R	Pilot observational clinical study	ForeSite PT pressure mapping system (XSENSOR technology Corp)	3 consecutive days (72 h)	Clinical/patient outcomes	Bed	Patients were turned less frequently than expected and 75% exceeded pressure thresholds > 10 times/day. CPM revealed risk patterns not otherwise detected, showing potential for care improvement.
Gammon, H M and Shelton, C B and Siegert, C and Dawson, C and Sexton, E and Burmeister, C and Gnam, G and Siddiqui, A	Prospective case steries	MAP System (Wellsense USA Inc), continuous bedside pressure mapping	4 h‐intervals up to 32 h plus	Clinical/patient outcomes	Bed	Patients were instructed to reposition. CPM recordings of 4 h‐intervals were assessed by an evaluator to determine if patients who were instructed to reposition did. This study showed they did. No HAPU developed during this time. Limitations—very mobile patients with low Braden scores—it is possible that these patients may have moved without education if they were uncomfortable. The study did not use CPM to determine if the repositioning made by the patient was effective.
Grap, Mary Jo and Munro, Cindy L and Wetzel, Paul A and Schubert, Christine M and Pepperl, Anathea and Burk, Ruth S and Lucas, Valentina	Descriptive, longitudinal study	Xsensor pressure mapping system (24 × 72 cell mat)	72 h continuously	Clinical/patient outcomes	Bed	Of the 132 subjects, 90.9% had no observed changes in skin integrity. Maximum interface pressure was above 32 mmHg virtually 100% of the time for the sacrum, left and right trochanter. At the 45 mmHg level, the left and right trochanter had the greatest amount of time above this level (greater than 95% of the time), followed by the sacrum, left and right scapula and the left and right heels. Similarly, at levels above 60 mmHg, the same site order applied.
Gunningberg, Lena and BÃ¥Ã¥th, Carina and Sving, Eva	Descriptive qualitative study	MAP System (Wellsense USA Inc), continuous bedside pressure mapping	Unknown	Biofeedback, clinical/patient outcomes	Bed	Focus groups and interviews to explore views on using CPM. Staff used CPM to prevent pressure injury in patients were unable to move themselves. They found it particularly useful when the number of the positions able to used for repositioning was limited to one or two positions. Also to reduce pressure to existing pressure damage. Useful if patients did not want to be turned overnight to determine if micro repositioning would help to relieve pressures without the patient waking up.
Gunningberg, Lena and Sedin, Inga‐Maj and Andersson, Sara and Pingel, Ronnie	Pragmatic RCT	MAP System (Wellsense USA Inc), continuous bedside pressure mapping	Max 14 days	Biofeedback, clinical/patient outcomes	Bed	Intervention consisted of randomly assigning patients to using CPM. PU status was collected at baseline and Day 3, 7 and 14. Interface pressures were measured. There were no significant differences in outcomes between the groups. Nursing staff stated they appreciated the feedback. The system does not have the ability to record data, therefore repositioning was based on real‐time rather than retrospective feedback.
Ho, C and Ocampo, W and Southern, D A and Sola, D and Baylis, B and Conly, J M and Hogan, D B and Kaufman, J and Stelfox, H T and Ghali, W A	Randomised controlled trial	Foresite PT CBPM system (Vista Medical Ltd.)	Continuous monitoring over 72 h	Biofeedback, clinical/patient outcomes	Bed	Intervention group had significantly reduced peak pressures and more frequent repositioning interventions. Nurses reported improved awareness and ease of use of CPM system. No difference in pressure injury incidence within 72 h.
Hubli, M and Zemp, R and Albisser, U and Camenzind, F and Leonova, O and Curt, A and Taylor, W R	Prospective cross‐sectional prepost pilot study	Sensomatic GmbH, Rothenburg Switzerland	3 weeks of continuous pressure monitoring	Biofeedback, clinical/patient outcomes	Wheelchair	All participants showed an increased relief frequency when using the feedback system, Friedman test showed statistical significance. Frequency and duration of pressure relief behaviour was improved in all participants. Relief behaviour reduced significantly when the feedback system was removed.
Hultin, L and A.‐C., Karlsson and Ohrvall, M and Gunningberg, L	Descriptive study	MAP system (Wellsense USA Inc), continuous bedside pressure mapping	Length of stay was between 5 and 52 days	Biofeedback, clinical/patient outcomes	Bed	CPM increased knowledge about PI and prevention. Visual feedback as an eye‐opener. Independence through participation in self‐care. Identify vulnerable pressure points. Changing position and getting control. The monitor provided visual feedback and information and increased knowledge of prevention.
Hultin, L and Olsson, E and Carli, C and Gunningberg, L	Descriptive—pre and posttest design	MAP system (Wellsense USA Inc), continuous bedside pressure mapping	7 consecutive days	Biofeedback, clinical/patient outcomes	Bed	A limited educational intervention, combined with the use of a pressure mapping system, was successful as it improved staff members' knowledge about PI prevention, reduced interface pressure and increased PI prevention activities. As many of the staff members lacked formal education in PI prevention and management, opportunities for teaching sessions and reflection upon PI prevention was recommended.
Motamedi, S. M. and De Grood, J. and Harman, S. and Sargious, P. and Baylis, B. and Flemons, W. and Ghali, W. A.	Pilot study; prospective interventional	XSENSOR ForeSite patient turn system (PTS)	Monitored throughout hospital stay (median 3 days, range 1–7 days)	Biofeedback, clinical/patient outcomes	Bed	Time in high‐pressure zones reduced from median 45% (baseline) to 8% (intervention) (*p* < 0.001). Number of effective repositioning events increased significantly during CPM feedback. Nurses reported improved decision‐making and awareness of patient positioning from real‐time data. Authors concluded that CPM with bedside display can substantially reduce high‐pressure exposure and may be an effective adjunct to standard repositioning protocols.
Ocampo W and Sola DY and Baylis BW and Conly JM and Hogan DB and Kaufman J and Kiplagat L and Stelfox HT and Ghali WA and Ho C	RCT + evalution study	xsensor pressure mapping system	Not clear	Biofeedback, clinical/patient Outcoomes	Bed	No results reported for the RCT. Surveys showed device is easy to use and improved how they provided pressure relief for their patients. Staff liked the turn timer. Some had negative feelings toward the system, namely the cost and inconvenience of settup up the device. It was felt by patients and carers that it influenced pressure ulcer prevention.
Sakai, Kozue and Sanada, Hiromi and Matsui, Noriko and Nakagami, Gojiro and Sugama, Junko and Komiyama, Chieko and Yahagi, Naoki	Observational study—development of PU	KINOTEX—Japan installed into the mattress Thermo Contour mattress—Barrington Healthcare	48 h	Clinical/patient outcomes	Bed	Area that showed the highest area of pressure was not always relieved—it was felt this was due to not using the biofeedback on the monitor to help to achieved.
Scott, R G and Thurman, K M	Interventional clinical study	MAP system (Wellsense USA Inc), continuous bedside pressure mapping	Not clear	Biofeedback, clinical/patient outcomes	Bed	Measured pressures were decreased when the CPM was used. Average peak pressure without CPM was 78 mmhg, when using CPM it was 47 mmhg. 100% of caregivers agreed they were more effective in repositioning with CPM. They also felt it increased quality of care. Families were less likely to refuse to be repositioned.
Tatsuta K and Sakata M and Sugiyama K and Kojima T and Akai T and Suzuki K and Torii K and Morita Y and Kikuchi H and Hiramatsu Y and Kurachi K and Takeuchi H	Prospective cohort study	Pressure and shear force sensors (Nissha Co. Ltd., Kyoto, Japan)	> 2 h	Clinical/patient outcomes	Operating table	Patients were divided into two groups according to the presence or absence of PI. PI had an incidence of 32.4% and the primary outcome, average horizontal shear stress, was significantly higher in the PI group than in the no‐PI group. The interface pressure increased over time in both groups. At 120 min, the interface pressure was two times higher in the PI group than in the no‐PI group (PI group, 221.5 mmHg; no‐PI group, 86.0 mmHg; *p* < 0.01).
Vos‐Draper TL and Morrow MMB and Ferguson JE and Mathiowetz VG	Longitudinal, repeated‐measures pilot intervention study	Vista medical BodiTrac pressure mat system with mobile pressure mapping app (mPMAP)	Total duration not reported; occurred during in‐clinic training session and intermittently at home over 1 month	Biofeedback, clinical/patient outcomes	Wheelchair	Confidence in weight shift effectiveness improved significantly with real‐time pressure map feedback (baseline 79.8 > 97.0 postfeedback). Confidence in preventing PI improved significantly after education alone (85.2 > 90.2), minor additional improvement postfeedback (94.3). Authors conclude real‐time CPM feedback improves confidence in effective pressure redistribution.
Woo, Kevin and Tran, Jake and Waters, Nicola	Prospective descriptive feasibility study	Smart sensor platform	1407 h of sensor monitoring (~13.5 h/patient avg)	Biofeedback, clinical/patient outcomes	Bed	Sensor system showed high agreement with nurse observations; strong feasibility for clinical use in pressure injury prevention and real‐time risk monitoring

### 3.3 Synthesis of Results

**FIGURE 2:**
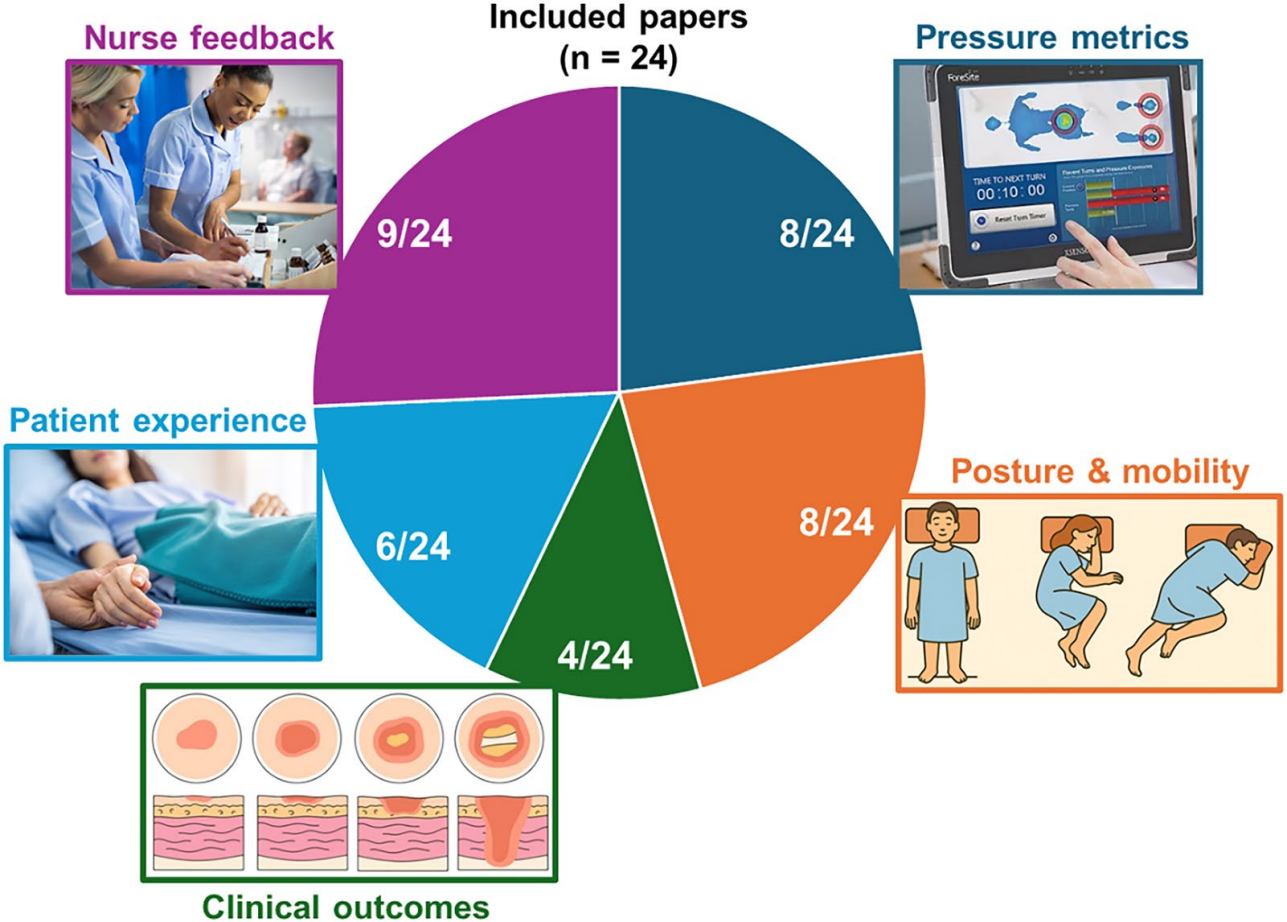
Distribution of the 24 included studies across five identified themes: Clinical outcomes, pressure metrics, posture and mobility, nurse feedback and patient experience.

Following review of the included papers, five core themes were identified: clinical outcomes [29, 31, 37, 41], pressure metrics [29, 30, 31, 33, 34, 41, 42, 45, 46], posture and mobility [19, 32, 35, 40, 42, 46, 47, 48], nurse feedback [17, 36, 37, 39, 43, 44, 47, 48, 50] and patient experience [17, 38, 42, 49, 50]. The distribution of studies across these themes is shown in Figure 2. Each theme was not mutually exclusive, with several papers contributing to more than one theme. These thematic groupings are explored in detail in the following sections of this review.

#### 3.3.1 Pressure Metrics

Several studies cite pressure values derived from the devices as important metrics to report in relation to PU risk. Some studies also used these values to compare or inform interventions. However, there was a high degree of heterogeneity in the measurement approach and reporting of pressure values. Across the literature, studies used a variety of parameters, including average pressure values [29], peak pressures [30, 34, 41] and regional analyses at specific anatomical sites [30, 33, 34, 41, 45]. However, justification for these thresholds was inconsistent, often referencing historic values such as 32 mmHg, derived from capillary closing pressures in nail‐fold studies, which have limited physiological transferability to deeper tissue sites such as the sacrum or heel [51].

Among studies that compared CPM intervention and control groups, findings were inconsistent. In a large, randomised trial, Ho et al. [29] found no significant difference between groups when using mean interface pressures or counts of sensels exceeding 40 mmHg, with most participants (> 99%) recording values above this threshold. Similarly, Grap et al. [41], used multiple thresholds (32, 45, 60 mmHg) in mechanically ventilated ICU patients and found that maximum sacral and trochanteric pressures were almost universally above 32 mmHg (> 99% of observations) and remained high at the upper thresholds, while mean pressures were far less frequently above these limits. These data highlighted the greatest sustained risk at the sacrum and trochanters, even under standard preventive care conditions.

By contrast, several intraoperative and experimental studies demonstrated reductions in localised peak pressures when CPM data were used to guide micro‐repositioning. Sving et al. [31] established a 40 mmHg risk threshold and observed a significant decrease in median sacral pressure during surgery when real‐time feedback was visible to staff compared with a blinded phase (37.9 vs. 44 mmHg, *p* = 0.001). Likewise, Al‐Majid et al. [45] and Tatsuta et al. [34] identified strong associations between procedural duration and sustained high sacral pressures, particularly during cardiac and colorectal surgeries, underscoring the cumulative risk from immobility. Other surgical and postoperative studies, including a textile‐based monitoring approach [30] and a fibre‐optic tactile sensor system [33], similarly linked extended high‐pressure exposure with tissue damage, particularly when repositioning was infrequent.

Across these studies, there was little consensus in the findings when comparing CPM intervention and control groups. While some demonstrated measurable reductions in localised pressures when CPM feedback was available [31], others found no significant differences using mean or peak values [29, 41]. Given the range of care settings studied, including acute wards, intensive‐care environments and surgical theatres and the diversity of reporting metrics, consistent evidence that CPM interventions can reliably reduce interface pressures remains limited.

Some studies explored patient characteristics influencing pressure exposure; body mass index (BMI) and surgical duration were linked as significant predictors of sacral interface pressure, with peak pressure increasing incrementally with each unit rise in BMI and with each additional minute of surgery [45]. Furthermore, another study included BMI as a risk factor to assess the correlation with the PUs injuries, reporting that both low (< 20.5) and high (> 32.5) BMI had a strong impact on pressure injuries risk [30]. These findings reinforce the multifactorial and patient‐specific nature of PI risk.

A more recent study by [46] introduced an intelligent algorithm to analyse long‐term CPM data from community‐dwelling individuals with PU. Rather than relying on static or mean pressures, this approach quantified cumulative exposure as the integral of pressure over time, defining risk categories based on both magnitude and duration of loading. Pressure–time thresholds, adapted from established sigmoid injury relationships, classified each posture into low, moderate, high or very high exposure levels (high risk > 45 mmHg/cm). Among 22 participants monitored for 5–47 h, many spent prolonged periods in static postures with high sacral pressures, while those who repositioned more frequently remained within lower‐risk zones. However, as with previous studies, the thresholds used to evaluate risk were still arbitrary and not tailored to the individual's clinical background or tissue tolerance.

#### 3.3.2 Posture and Mobility

Several studies used the data from CPM devices to track posture and mobility parameters. This included both characterising mobility as a risk score and using the data to support position changes or repositioning advice.

Various studies have explored analytical and sensing methods to interpret CPM data for posture classification. ML approaches have been particularly effective. For instance, Caggiari et al. [19, 46] and Fryer et al. [47] used derivatives of pressure metrics such as gradients and rates of change to detect both large and subtle movements. Caggiari et al. [46] further introduced sigmoid modelling to describe how pressure evolves over time, quantifying posture duration and integrated convolutional neural networks (CNNs) that classified static postures with 70%–84% accuracy. In community and clinical settings, their CPM systems detected large‐scale movements based on changes in centre of pressure and contact area, revealing that many participants spent over 60% of the monitoring time in static postures exceeding 2 h. While Fryer et al. [47] focused on identifying *Movements to Offload Vulnerable Areas* (MOVAs) in spinal cord injury (SCI) patients and found strong correlations between algorithm predictions and nursing observations. Movement frequency and timing were significantly influenced by injury level, with high‐level SCI (C1–T6) patients showing fewer movements and longer intervals between off‐loading events up to 10 h while lying and 6 h while sitting.

Other researchers have adopted threshold‐based and multisensor approaches [35]. For instance, Woo et al. [35] combined CPM with temperature and moisture sensors to provide comprehensive PU risk assessment, achieving 95.7% agreement with nurse evaluations and enabling real‐time visualisation of pressure, temperature and humidity for early detection of high‐risk conditions.

Expanding beyond detecting movement patterns, CPM systems have also been used to encourage movement and repositioning through real‐time feedback. In both community [40, 42] and hospital settings [32, 48], this feedback was directed either to patients or to clinical staff and consistently improved mobility behaviour; among wheelchair users with SCI, two studies (Chenu et al. [42] and Hubli et al. [40]) showed that seat‐mounted pressure mapping systems providing tactile or mobile feedback prompted more frequent pressure reliefs and reduced long periods of static sitting. Hubli et al. [40] reported the frequency of pressure reliefs rose from 11% to 82% while feedback was active, though this effect declined once feedback was withdrawn. In hospital bed settings, Motamedi et al. [48] discovered that by displaying real‐time pressure maps to nurses, a significant increase in assisted turns and mobility events was observed, while a similar study by Gammon et al. [32] confirmed that > 98% of patients capable of self‐turning repositioned effectively and none developed pressure injuries. Collectively, these studies show how CPM can act not only as a monitoring tool but also as a means of guiding preventive mobility in daily care, providing clinicians with actionable insight and empowering patients to take an active role in their own repositioning.

#### 3.3.3 Clinical Outcomes

There were studies which assessed whether employing CPM in care settings would reduce the incidence of PUs. This includes randomised controlled trials (RCT), prepost CPM intervention studies and observational cohort studies. Studies presenting clinical outcomes typically involved nurse‐led assessments of skin integrity and classified PUs using guidance from various global PI advisory bodies [52], or the current unified guidelines published by the European Pressure Ulcer Advisory Panel (EPUAP) [53].

Two studies used RCT methods [29, 37], typically using pragmatic designs to investigate whether the use of CPM could reduce the incidence of pressure injuries. Of these hospital‐based studies (Ho et al. [29] and Gunningberg et al. [37]), patient groups were assigned either to receive CPM visual feedback displayed to staff or to a control condition where the monitor was concealed, while all participants received standard preventive care, including repositioning and the use of pressure‐relieving mattresses. In both studies, there was no significant difference in the incidence of new PUs between the intervention and the control groups (*p* > 0.05). It is of note that trial design varied, with monitoring periods ranging from 72 h in acute care [29] to 14 days in geriatric wards [37], with all using an international staging system (NPIAP categories 1–4). Gunningberg et al. [37] also reported that while all patients received standard preventative interventions (e.g., foam or air mattresses, turning schedules, heel cushions), the latter were applied more frequently in the control group at Day 14 (28 vs. 15, *p* = 0.033), possibly reflecting greater reliance on conventional measures when CPM feedback was unavailable. Furthermore, the researchers noted unanticipated data loss, given that CPM generates extremely large datasets that can strain hospital servers, thereby limiting the study's statistical power and the interpretability of its findings [37]. Subgroup analysis from Ho et al. [29] demonstrated that among participants with a pre‐existing PI, those in the intervention group developed fewer new ulcer sites compared with controls (10.5% vs. 26.1%, *p* = 0.004). This effect was evident in the nonintensive care unit (ICU) subgroup (5% vs. 22%, *p* = 0.005) but not among ICU patients.

Observational cohort studies, both in critical or perioperative care, reported that most patients (86.0%–90.9%) maintained stable skin integrity during CPM monitoring, with new pressure injuries being uncommon when preventive practices and CPM‐guided repositioning were consistently applied [31, 41]. Only 5.3% of ICU patients (*n* = 132) [41] and 13%–14% of perioperative patients experienced skin change during the intervention (*n* = 43) and control phases (*n* = 43 and 30) [31]. Perioperative cohorts did not report any new PUs even after procedures lasting up to 5 h; transient blanchable erythema was only observed postsurgery, resolving within 24 h [31]. Pressure‐related tissue changes were typically preceded by sustained exposure to high pressures, particularly within the sacral region [31, 41]. Collectively, the findings from both controlled and observational studies indicate that CPM can help target high‐risk areas and supports early detection of pressure‐related changes. However, CPM implementation has yet to demonstrate a clear reduction in ulcer incidence beyond that achieved through standard repositioning and support‐surface protocols.

#### 3.3.4 Nurse Feedback and Patient Experience

In addition to the quantitative outcomes cited above, studies have also explored the qualitative feedback from users of CPM devices, including patients, carers and healthcare professionals. These study methodologies have provided an in‐depth understanding of user experience and highlighted some of the key ways in which CPM devices support shared decision making and understanding of PU risk.

The majority of included studies were conducted in acute hospital settings [36, 38, 44, 48, 50] with similar results reported in care home and community contexts [17, 39, 42, 49]. Study designs were predominantly descriptive, employing semi‐structured interviews, focus groups, questionnaires or mixed‐methods approaches. Perspectives were gained from patients ranging from independently mobile hospitalised adults to individuals with severe mobility limitations, including wheelchair users. Patient acceptability of CPM was generally high. Over half of patients reported the system to be comfortable, with only a small minority requesting removal. Approximately 52.7% of respondents agreed or strongly agreed that CPM influenced pressure injury care, while 25% of patients and family members perceived a positive impact on care quality [50].

Across several studies, CPM was reported to enhance healthcare professionals' confidence in repositioning patients and interpreting interface pressure risk [36, 44, 50]. One nurse was cited:

‘Before you walked around and checked that they were sleeping, that they were breathing, and that everything was calm. Now you walk around and check that, plus you check the monitor. That's a part of the actual procedure’.

However, change among staff was inconsistent; some nurses reported that CPM did not significantly alter established care routines. Limited training was identified as a contributory factor, with one physiotherapist stating:

‘I haven't actually used the monitor. I didn't receive enough information about what I should do so I haven't used it. I've just done the same as always’ [36].

Education on CPM appeared to be a key factor. Hultin et al. [38, 39] demonstrated that even brief educational interventions, when combined with CPM, improved staff knowledge of pressure injury prevention, reduced interface pressures and increased preventive activities. Reporting that in a long‐term care facility, where a 35‐min training session led to measurable improvements in knowledge and practice.

Reported disadvantages from staff included cost considerations and challenges associated with the application of the technology to a patient's mattress. Concerns were also reported that patients or carers could become overly focused on the monitor. Some nurses questioned the accuracy of the colour displays, particularly when images remained predominantly blue despite prolonged static positioning. As one nurse explained:

‘Some days it's blue all the time, even though the patient hasn't moved’ [48].

In contrast, multiple studies identified the empowering effect of real‐time colour feedback for both patients and carers, particularly older hospitalised adults [36, 38, 39, 44, 50]. Patients frequently described CPM as an ‘eye‐opener’, enabling them to recognise pressure points and make small independent adjustments. One 70‐year‐old participant stated:

‘I never realised that all I had to do to avoid getting pressure sores was to adjust my body position a tiny bit’ [38].

This increased independence reduced reliance on nursing staff and supported patient engagement in preventive care. As one older patient explained:

‘It really doesn't take a lot to adjust your own body position—you don't need to call an attendant and make a fuss just to get yourself turned over’ [38].

Qualitative feedback highlighted reassurance and increased confidence in PU self‐management, with one carer remarking:

‘This is amazing and has given me control and stopped the guesswork’ [17]

and another noting how family members engaged with the display:

‘Grandchildren were very interested and kept saying ‘don't worry, there are no red spots’ [17].

However, participants also highlighted technical limitations, including the absence of alarms or reminders and the lack of data recording functionality. These limitations restricted clinicians' ability to assess repositioning frequency, adherence and longer‐term behaviour change [38].

## 4 Discussion

The scoping review revealed that CPM has been used in a variety of clinical settings to monitor, assess and inform interventions for the prevention and treatment of PUs. There was a high degree of heterogeneity in the patient populations, application time of the CPM and interpretation of the data which is derived from the commercial systems. The literature was thematically described in five domains: pressure distribution and mobility data, qualitative insights relating to patient comfort and acceptability, perceptions of healthcare staff regarding usability and clinical integration of the technology. Community settings were under‐represented, limiting insight into how CPM performs in everyday care environments where prolonged immobility is most common [54]. Furthermore, few mixed‐methods approaches were employed, reducing understanding of how CPM influences practice, decision‐making and patient experience. The translation of these findings was limited by the predominance of small‐scale studies and the lack of consensus in outcome measures appropriate to assess the efficacy of CPM for PU prevention and treatment.

Studies employing CPM reported pressure data using a wide variety of metrics, with little consistency in definitions or application. This includes simplified metrics of average or peak pressures and the use of thresholds above which patients were deemed at higher risk. For example, 40 mmHg was used as a cut‐off [29] adopted from a small pilot study that did not itself report quantitative metrics [48], or studies acknowledging that other publications use higher threshold values [41]. Average pressures create redundancy across both anatomical regions and monitoring duration, masking localised hotspots and transient fluctuations that are critical for PU risk. As a result, mean values fail to capture the combined influence of magnitude, duration and distribution, which may explain inconsistent associations with clinical outcomes.

This lack of consensus underscores that there is no universal ‘safe pressure’, as pressure tolerance is patient‐specific and influenced by soft tissue properties, comorbidities and history of pressure injury. Pressures values from the CPM studies routinely were within levels cited to cause partial tissue ischemia compromise (≥ 32–60 mmHg) [51], with exposure often linked to surgical duration and posture [31, 45]. Intraoperative work illustrates how real‐time CPM feedback can support micro‐adjustments when patients are immobile and vulnerable, but such benefits were offset by limitations including short follow‐up (typically 1 day), nonrandomised designs and heterogeneity in reporting pressure metrics, even when the same monitoring system was used. Together, these factors highlight the need for standardised and clinically relevant ways to interpret long‐form CPM data. To address the importance of time dependent data, Caggiari et al. [46] used a sigmoid model that integrates both the magnitude and the duration of pressure exposure, and categorised the risk based on the cumulative loading rather than static values. This model with their intelligent algorithms offered interpretation of CPM data by linking the posture and the exposure time to an established sigmoid risk relationship [46]. However, it still relied on fixed thresholds, which still does not accommodate the personalised nature of tissue tolerance to mechanical loads.

Studies have shown that patients usually spend a long time in static postures, often exceeding 2 h, even when following repositioning protocols, which highlights that immobility remains a key issue that leads to tissue damage [55]. Advanced CPM technologies have enabled more detailed data of movement behaviour and can influence nurses' decision‐making. In addition, the integration of intelligent algorithms with CPM can provide automated posture detection and classification, validated against gold standard wearable actimetry sensors [20]. However, generally, existing studies are still limited by small sample sizes, reliance on generalised pressure thresholds instead of personalisation and clinical trials. Furthermore, there is still a lack in the integration of CPM with healthcare systems, for instance, the variations in pressure relieving mattresses or cushions and random postures may affect the output data from CPM and the algorithm sensitivity.

Studies reporting clinical outcomes with CPM have primarily presented the incidence of PUs, often without detailed information on wound characteristics and changes over time [29, 31, 41]. An exception was one trial that undertook repeated skin assessments at several timepoints, classifying PUs by severity category, which provided a more transparent picture of ulcer progression [37]. Such detailed and standardised reporting should be encouraged, as it enables critical appraisal of whether CPM‐informed interventions translate into meaningful clinical benefit. From the studies included in the review, the evidence for CPM reducing ulcer prevalence or incidence remains limited, with the two published RCT reporting no significant differences in new ulcer development between intervention and control groups [29, 37].

A further issue lies in how CPM data are used by the clinical team. Indeed, studies have shown that nursing staff are often unaware of the actual effectiveness of their repositioning interventions in offloading tissue, a gap that may be exacerbated if CPM data are not fully understood [56, 57]. Feedback displays provide valuable information, but if the data are not interpreted correctly, their preventative potential may be lost. Improving training and embedding ‘skin champions’, such as tissue viability nurses with expertise in CPM technology, may help bridge this gap and ensure the technology is applied effectively in practice. Collectively, these studies underline that while CPM represents the most advanced noninvasive method for measuring skin–support surface interface pressures at the bedside, the effectiveness depends not only on the accuracy of the technology but also on clinical integration, staff education and consistent reporting of outcomes.

Generally, it was reported that CPM improved staff awareness, patient repositioning and PU prevention activities in hospitals, care homes and community settings. Short educational interventions combined with CPM enhanced knowledge and confidence in pressure management. Several studies used technology which lacked the ability to integrate data due to a lack of recorded data, resulting in an inability to review data retrospectively. However, while patients noted the absence of reminders and alarms, alerting systems that are frequent, nonspecific or poorly aligned with clinical workflows may contribute to alarm fatigue and reduce staff responsiveness [58]. This highlights the need for CPM alerts that notify the need for targeted, clinically meaningful intervention, while mitigating false or nonactionable alarms.

The surveys and qualitative studies concentrated on whether their healthcare worker responded well to the colour image rather than whether the patient responded to the colour image. Overall, the volume of qualitative data was limited, with relatively sparse feedback from both patients and clinicians. It was noted, however, that healthcare workers felt patients became fixated on the image; this was not explored as to why this might be. In some cases, nurses reported inaccuracies in the visual feedback, resulting in a lack of confidence in the system; however, in other cases, it was felt that the visual image supported the novice clinician to make an informed decision when repositioning. It was felt by one of the research teams that they may have been too optimistic when introducing the system, and it is possible that staff did not use the information regarding pressure points from the CPM monitor in bedside nursing care as expected [37]. This is notable given that biofeedback has been shown to be an effective approach across a range of healthcare disciplines, particularly in rehabilitation and chronic condition management, where augmented visual or sensory feedback can support behaviour change, motor retraining and symptom control [59].

### 4.1 Limitations

This review focused on studies that applied CPM systems in different care settings, with the intention of providing a comprehensive overview of real‐world applications across diverse care environments. While this approach maximised the inclusion of clinically relevant evidence, it inherently excluded experimental and preclinical studies reporting advances in sensor technologies that have not yet been evaluated in patients. For more detailed information on recent developments in pressure sensor design and performance with potential for clinical translation, reference can be made to the technically oriented review by van Helden et al. [16], which complements the scope of the present work.

Only studies published in English were included, which may have introduced language or publication bias. Although multiple databases were searched using a broad strategy to capture relevant literature, some eligible studies may not have been retrieved, particularly unpublished or grey literature. Furthermore, due to the heterogeneity of study designs, patient populations and outcome measures, quantitative synthesis through meta‐analysis was not feasible; consequently, findings were narratively summarised. Most studies employed commercial pressure mapping systems, including the XSensor ForeSite PT and OR systems (XSENSOR Technology Corp, Canada) and the MAP System (Wellsense, USA), which produce data at terabyte scale over prolonged monitoring. Manufacturers of these systems need to prioritise streamlining integration with existing healthcare servers, requiring optimisation of sensor resolution and sampling rates without losing clinically relevant information.

Future improvements in the technology should seek seamless integration with electronic health records and support surfaces to facilitate automated auditing, clinical decision‐making and continuity of care. There is an inherent need to establish standard frameworks for reporting patient outcomes and pressure metrics, which would enable meaningful comparison across studies and care settings. Clear guidance materials and well‐organised staff training programmes also need to be developed to improve the usability of CPM systems and embed the technology into routine clinical practice; co‐developing with clinicians, patients and caregivers may help adapt CPM technologies to real‐world care environments and to the needs of the end user, while also promoting self‐management and patient empowerment [60]. Finally, comprehensive health economic evaluations are required to assess cost‐effectiveness and sustainability at scale. Progress in these areas will be key to establishing CPM as a reliable, data‐driven component of personalised PU prevention.

## 5 Conclusion

This review synthesised evidence on the use of CPM in diverse clinical environments. Collectively, the studies suggest CPM may enhance awareness of interface pressures, aid in clinical decision‐making and inform repositioning techniques for those who are at risk of tissue injury. Increased staff confidence and patient engagement are among the reported benefits. However, consistent reductions in ulcer incidence have yet to be demonstrated, which could be due to the technology being limited to monitoring the mechanical interactions with support surfaces. Indeed, these pressures may not directly relate to tissue deformation known to cause PUs. Further studies are required to refine the implementation of CPM, assess its efficacy to prevent or treat PUs, and evaluate whether it represents a cost effective technology for use in care settings.

## Funding

This work was supported by the Engineering and Physical Sciences Research Council (EP/W031558/1).

## Ethics Statement

The authors have nothing to report.

## Conflicts of Interest

The authors declare no conflicts of interest.

## Data Availability

The data that support the findings of this study are available on request from the corresponding author. The data are not publicly available due to privacy or ethical restrictions.
